# Prognostic Impact of Tumor-Associated Macrophage-Related Markers in Patients with Adenocarcinoma of the Lung

**DOI:** 10.1245/s10434-023-13384-9

**Published:** 2023-06-06

**Authors:** Shunsuke Shikanai, Noriyuki Yamada, Naoki Yanagawa, Mayu Sugai, Mitsumasa Osakabe, Hajime Saito, Makoto Maemondo, Tamotsu Sugai

**Affiliations:** 1 Department of Molecular Diagnostic Pathology, School of Medicine, Iwate Medical University, Shiwagun’yahabachou, Japan; 2 Department of Respiratory Medicine, School of Medicine, Iwate Medical University, Shiwagun’yahabachou, Japan; 3 Department of Thoracic Surgery, School of Medicine, Iwate Medical University, Shiwagun’yahabachou, Japan

## Abstract

**Background:**

Macrophage polarization is an important pathogenetic factor in neoplastic diseases. Phosphorylated signal transducer and activator of transcription 1 (phospho-STAT1) regulates the M1 phenotype, and c-Maf regulates the M2 phenotype. However, the role of macrophage phenotype in lung adenocarcinoma (LAD) remains unclear.

**Patients and Methods:**

We examined whether the density of M1 and M2 macrophages was associated with prognosis in patients with LAD using double-labeling immunohistochemistry. In addition, programmed death ligand 1 (PD-L1) expression was investigated. Immune cells coexpressing CD68 and phospho-STAT1 were considered M1 macrophages, whereas those coexpressing CD68 and c-Maf were recognized as M2 macrophages. Patients with LAD (*N* = 307) were divided into two cohorts (*n* = 100 and *n* = 207) to evaluate the associations of M1 and M2 phenotypes with prognosis in patients with LAD. We determined the cut-off values of CD68/phospho-STAT1-positive cells and CD68/c-Maf-positive cells to assess correlations with overall survival (OS) using receiver operating characteristic curve analysis in the first cohort.

**Results:**

According to the cut-off values of 5 or less CD68/phospho-STAT1-positive cells and more than 11 CD68/c-Maf-positive cells, high expression of CD68/c-Maf and low expression of CD68/Phospho-STAT1 were identified as independent prognostic markers for OS and disease-free survival (DFS). Moreover, the M1/M2 ratio (0.19 or less) was a poor prognostic factor for OS and DFS. However, PD-L1 expression did not correlate with patient outcomes.

**Conclusions:**

Overall, these findings suggest that double immunostaining of markers of phospho-STAT1 (M1) and c-Maf (M2) can be used as prognostic indicators for patients with LAD.

**Supplementary Information:**

The online version contains supplementary material available at 10.1245/s10434-023-13384-9.

Lung cancer is the most common cancer worldwide and the leading cause of cancer-related death.^1,2^ The underlying molecular mechanisms of lung carcinogenesis are complex.^3,4^ The tumor microenvironment has attracted much attention in the study of solid tumors,^4,5^ and tumor cells and surrounding stromal cells play important roles in tumor progression and cancer metastasis.^5,6^ The link between tumor and stromal cells may be associated with tumor growth and progression.^5,6^ Stromal cells include fibroblasts, vascular cells, and immune cells;^5,6^ common immune cells, including lymphocytes, neutrophils, and monocytes/macrophages, are also involved in the tumor microenvironment.^5,6^ Among these, macrophages are major players in tumor progression and metastasis.^7–11^

Macrophages are heterogeneous and can be polarized into proinflammatory M1 macrophages (classical type) or anti-inflammatory M2 macrophages (alternative type).^11,12^ Both phenotypes are involved in tumor-related inflammation, whereas M2 macrophages promote angiogenesis, wound healing, and tumor growth.^11,12^ In the absence of M1 macrophage-orienting signals, M2 macrophages promote tumor cell proliferation in vitro and in preclinical models; evaluation of distinct protumor and antitumor macrophage subsets is a challenging research topic.^13,14^

Previous studies have provided conflicting evidence regarding the significance of macrophages in cancer.^15,16^ In colorectal tumors, infiltrating macrophages have pro-inflammatory properties and consequently play antitumor roles.^15,16^ Thus, some macrophage types are associated with a good prognosis.^15,16^ However, other studies have shown that macrophages promote tumor progression and are associated with poor prognosis in lung, cervical, ovarian, esophageal, and breast cancers, as well as melanoma.^17,18^ After recruitment to the tumor site, macrophages are polarized into a specific phenotype (tumoricidal or tumorigenic) via exposure to tumor microenvironment-derived factors, such as cytokines, growth factors, and hypoxia.^15–18^ According to this theory, immune clearance of solid tumors, which is induced by macrophages, is altered by various factors.^15–18^

The role of macrophages in lung adenocarcinoma (LAD), the most common histological type of lung cancer, remains unclear. Therefore, we evaluated stroma-infiltrating macrophages (M1 and M2 phenotypes) and investigated the associations of M1/M2 macrophages with prognosis in patients with LAD. In addition, programmed death ligand 1 (PD-L1) expression was examined in tumor cells and tumor-infiltrating immune cells.

## Patients and Methods

### Patients

In total, data from 307 patients with LAD who were treated at Iwate Medical University between 2010 and 2016 were analyzed in the present study. Tumor histological classifications were determined according to the World Health Organization (WHO) classification criteria.^3,4^ Tumors were graded on the basis of International Association for the Study of Lung Cancer/American Thoracic Society/European Respiratory Society (IASLC/ATS/ERS) classifications.^19^ Classification was according to the predominant histologic pattern, which often coexists with less dominant elements of other histologic patterns.^20^ We defined tumor spread through air spaces (STAS) as micropapillary or solid clusters of single tumor cells floating free within air spaces beyond the edge of the tumor, as previously reported.^21^ With the IASLC classification, lung adenocarcinoma (LAD) was classified into four categories: (1) preinvasive lesion, including adenocarcinoma in situ, (2) minimally invasive LAD, (3) invasive LAD, and (4) variants. The architectural grading system, which is the system most frequently referenced, is based on the predominant growth pattern of the invasive adenocarcinoma. It contains the following three grades: (1) low (minimally invasive LAD), (2) intermediate (lepidic adenocarcinoma, acinar and papillary adenocarcinoma), and (3) high (solid, micropapillary adenocarcinoma and invasive mucinous adenocarcinoma). Tumor-infiltrating lymphocytes were defined as described elsewhere.^22,23^ Histological specimens were fixed within 1 h after resection, as phosphorylated proteins can be rapidly dephosphorylated if the specimen is not immediately fixed in formalin.

The Ethics Committee of Iwate Medical University School of Medicine approved this study (approval no. MH2021-068). All patients provided written informed consent to participate, according to institutional guidelines. All study protocols were performed according to the standards set by the Declaration of Helsinki.

### Determination of Overall and Disease-Free Survival

The follow-up period was 1825 days. Lung cancer-specific survival, defined as cause of death from lung cancer, was used to assess overall survival (OS). In addition, recurrence-free survival, which excluded secondary cancers, was adopted to measure disease-free survival (DFS). The duration of disease-free survival was determined on the basis of the presence/absence of metastasis evaluated during the follow-up period (three to four times per year) with computed tomography.

### Sample Size Determination

The required sample size was determined to be at least 100 cases (for cohort 1, the development cohort) using the pROC package via R statistical software. The statistical power (detection power) was set to 0.9, which is commonly used in medical studies.

### Chemotherapy After Surgery for LAD

Following surgery, tegafur uracil was administered to 85 of the 165 patients with LAD who received postsurgical treatment, tegafur/gimeracil/oteracil was administered to 1 patient, and platinum-doublet chemotherapy, including carboplatin plus gemcitabine, tegafur/gimeracil/oteracil, or paclitaxel, was administered to 62 patients. Radiotherapy was performed on 12 patients, and tyrosine kinase inhibitors were administered to 5 patients. The other 142 patients did not receive additional chemotherapy following surgery. Therefore, the present study included only patients who were treatment-naive before surgery. In addition, no patient received immunotherapy in the present study.

### Construction of Tissue Microarrays (TMAs)

One core per tumor was obtained in the present study. Paraffin-embedded tissues used for construction of TMAs were stored at room temperature. We created TMAs using a manual tissue array (Azumaya Co., Tokyo, Japan). We selected invasive areas with an absence of basement membrane, as confirmed by elastic Masson stain, for immunohistochemical assessment. In addition, the selected areas contained massive infiltrates of immune cells. We excluded alveolar macrophages, which are often present in airspaces entrapped in tumor areas, and consequently, only stromal macrophages in areas of invasion were counted. A lepidic pattern was not contained in the TMAs, given that such a pattern is not found in invasive areas.

Invasive areas were identified by expert pathologists (N.Y. and T.S.). Three-millimeter-thick tissue cores were obtained from target lesions and placed into recipient blocks containing 12 cores, including 10 cancer tissues and 2 control tissues. After construction, we prepared 3-μm-thick sections stained with hematoxylin and eosin (HE) using the initial slides to confirm the histological diagnosis. Serial sections were cut from TMA blocks for immunohistochemical staining.

### Immunohistochemistry for PD-L1 (Dako, Clone, 22C3)

Three-micron-thick tissue sections were placed on charged slides, dried, and melted at 42 °C for 180 min. After deparaffinization and rehydration, the sections were heated in Envision FLEX target retrieval solution (pH 6.0 or 9.0, Dako) for 20 min and washed twice for 5 min in phosphate-buffered saline. Hydrogen peroxide (3%) was used to block endogenous peroxidase activity for 5 min. Immunohistochemistry was performed using the Dako Envision+ system. The specimens were heated in citrate buffer (pH 6.0) for 20 min each using PT Link (Dako). The antigen-antibody reaction was visualized using an enhanced polymer-based detection system. Hematoxylin was the counterstain. Sections of invasive colorectal cancer and interstitial lung disease were used as positive controls.

### Double Staining Immunohistochemistry

Blocks were sectioned at a thickness of 3 μm. Antibodies targeting CD68 (clone PG-M1, Dako), phosphorylated signal transducer and activator of transcription 1 (phospho-STAT1; monoclonal, clone, and 58D6, Cell Signaling Technology, Danvers, MA, USA), and c-Maf (clone EPR16484, Abcam, Cambridge, UK) were used for analyses. Double staining was performed using a Dako Envision+ system with dextran polymers conjugated with horseradish peroxidase (Dako), as previously described.^12^ First, sections were stained with anti-CD68 antibodies for 30 min at room temperature, generating a brown color. Denaturing solution (BioCare Medical-CA, USA) was added for 5 min at room temperature for elution during double staining. Antigen retrieval was performed by heat treatment for 45 min with HIER T-EDTA Buffer (Dako). After incubation, sections were reacted with phospho-STAT1- or c-Maf-specific reagents using dextran polymers conjugated with horseradish peroxidase (Dako) overnight at 4 °C, using a Vina Green Chromogen Kit (BioCare Medical-CA), which produced green staining. Finally, slides were washed in Wash Buffer (Dako) for 3 min. Sections were counterstained with hematoxylin. The antibodies used in this study are listed in Supplementary Table 1.

### Assessment of Immunohistochemical Results

We examined the immunohistochemical expression of CD68/c-Maf and CD68/phospho-STAT1 using whole sections to evaluate the heterogeneous expression of these markers. Although heterogeneous expression was found within cancer tissue to some extent, ten fields that contained immune cells within the invasive area were determined to avoid differences in heterogeneous positivity of the immune-stained cells by visual assessment. Those ten fields were selected as invasive areas with massive infiltrates of immune cells, because tumor-associated macrophages (TAMs) were infrequently detected in strong desmoplastic reaction areas. As a result, the immunopositivity of TAMs was examined for CD68/c-Maf and CD68/phospho-STAT1. Inflammatory cells were carefully excluded from analysis. Only nuclear positivity for c-Maf and phospho-STAT was considered significant, whereas only cytoplasmic expression of CD68 was regarded as positive. The immunostaining intensity (weak, moderate, and strong) was evaluated, and positive cells were defined as those showing greater than moderate intensity (moderate and strong staining). Positive cells were counted in the ten selected fields, and the total number of positive cells was determined. In addition, sections showing greater than 1% PD-L1 immunohistochemical expression in the tumor and tumor-infiltrating immune cells were considered positive. Determination of positive cells was performed by expert diagnostic pathologists (S.S., N.Y., and T.S.) blinded to the study endpoint. If results among the pathologists were discordant, a consensus was reached through discussion.

### Statistical Analysis

Data were analyzed using JMP Pro 16.1 software (SAS). Fisher’s exact tests were used to compare differences in clinicopathological findings between cohorts. Age distributions were compared using Mann–Whitney *U* tests. Kaplan–Meier analyses were performed using log-rank tests for survival analyses. Univariate and multivariate analyses were conducted using Cox proportional hazards models to identify variables predicting overall and disease-free survival. Significance was set at *p* < 0.05, and 95% confidence intervals were determined.

## Results

In total, 307 patients were analyzed. Based on the 8th edition of the American Joint Committee on Cancer Staging Manual, 197 patients had stage I disease, and 110 patients had stages II and III disease. Histopathological variables, including lymphatic invasion, venous invasion, pleural invasion, and STAS, are summarized in Table 1. Patients were divided into two cohorts, including the first (for cut-off values to evaluate patient survival) and second cohorts (validation). We used a block randomization method in the research design to select and divide participants into different groups or conditions to avoid bias in the selection of the two cohorts. There were no significant differences in clinicopathological findings between cohorts. Clinicopathological variables are summarized in Table 1.

**Table 1:** Clinicopathological findings in patients with lung adenocarcinoma

Factor	Cohort 1		Cohort 2		*p* Value
Total	100		207		
Age (years), median (range)	70	(45–88)	70	(40–88)	NS
Sex					NS
Male (%)	47	(47.0)	97	(46.9)	
Female (%)	53	(53.0)	110	(53.1)	
Smoking					NS
Smoker (%)	53	(53.0)	100	(48.3)	
Never (%)	47	(47.0)	107	(51.7)	
pStage					NS
I (%)	71	(71.0)	126	(60.9)	
II (%)	16	(16.0)	32	(15.5)	
III (%)	13	(13.0)	49	(23.6)	
IASLC/ATS/ERS classification subtypes					NS
Low group (%)	6	(6.0)	15	(7.3)	
Intermediate group (%)	74	(74.0)	152	(73.4)	
High group (%)	20	(20.0)	40	(19.3)	
Lymphatic invasion					NS
Positive (%)	23	(23.0)	36	(17.4)	
Negative (%)	77	(77.0)	171	(82.6)	
Venous invasion					NS
Positive (%)	21	(21.0)	42	(20.3)	
Negative (%)	79	(79.0)	165	(79.7)	
Pleural invasion					NS
Positive (%)	31	(31.0)	54	(26.1)	
Negative (%)	69	(69.0)	153	(73.9)	
STAS					NS
Positive (%)	31	(31.0)	67	(32.4)	
Negative (%)	69	(69.0)	140	(67.6)	
Postsurgical therapy					NS
Chemotherapy (%)	51	(51.0)	102	(49.3)	
Radiation therapy (%)	3	(3.0)	9	(4.3)	
None (%)	46	(46.0)	96	(46.4)	
Recurrence					NS
Positive (%)	37	(37.0)	88	(42.5)	
Negative (%)	62	(62.0)	113	(54.6)	
Unknown (%)	1	(1.0)	6	(2.9)	
Survival status					NS
Alive (%)	66	(66.0)	131	(63.3)	
Dead (%)	34	(34.0)	76	(36.7)	
Disease-free survival (days), median (range)	1825	(87–1825)	1651	(53–1825)	NS
Overall survival (days), median (range)	1825	(87–1825)	1800	(95–1825)	NS

*NS* not significant, *pStage* pathological stage, *IASLC* International Association for the Study of Lung Cancer, *ATS* American Thoracic Society, *ERS* European Respiratory Society, *STAS* spread through air spaces

**Fig. 1:**
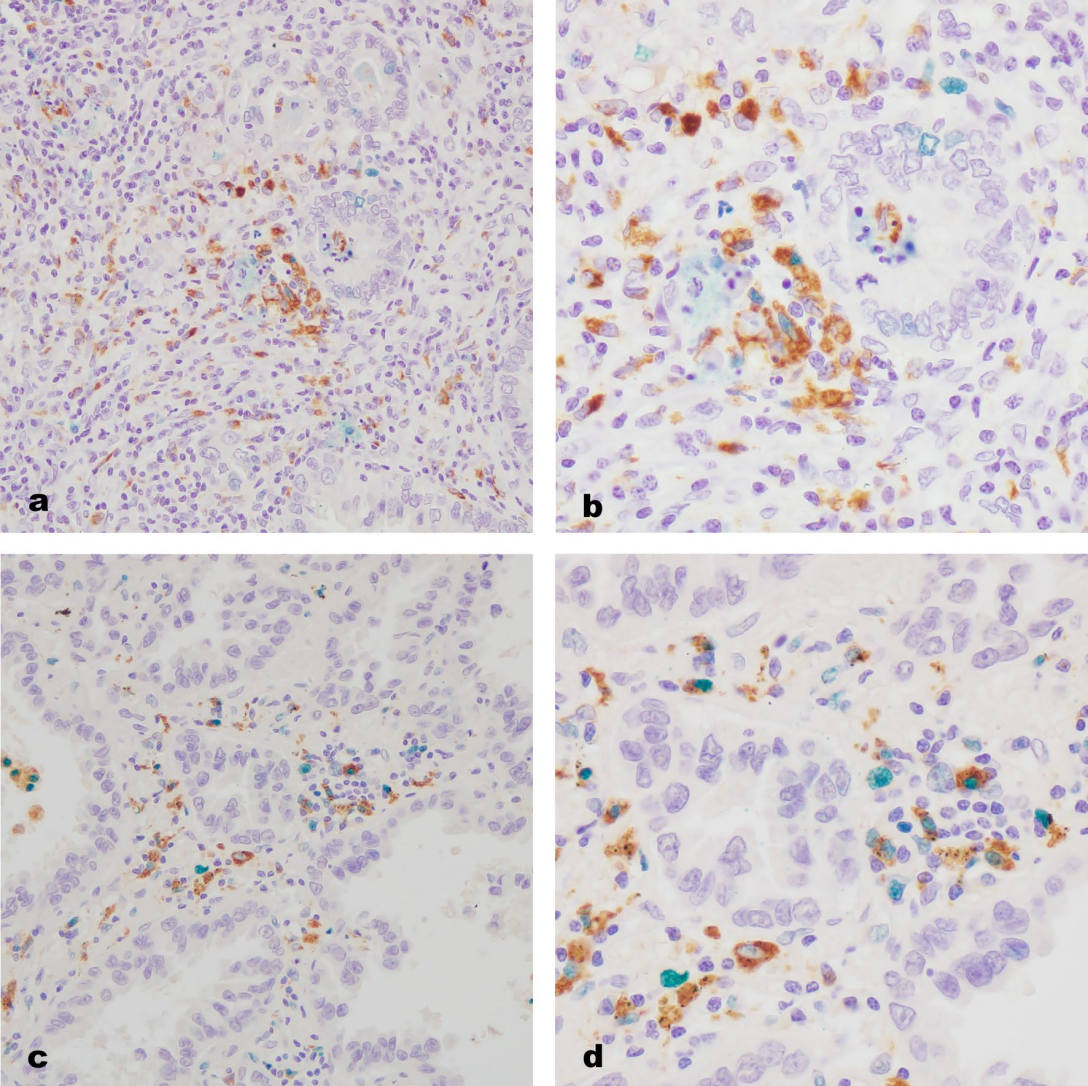
Double immunostaining of CD68/phospho-STAT1 and CD68/c-Maf in massive infiltrates of immune cells. **a** CD68/phospho-STAT1-positive macrophages (brown, CD68; green, phospho-STAT1), low-power field (×20). **b** CD68/phospho-STAT1-positive macrophages (brown, CD68; green, phospho-STAT1), high-power field (×40). **c** CD68/c-Maf-positive macrophages (brown, CD68; green, c-Maf), low-power field (×20). **d** CD68/c-Maf-positive macrophages (brown, CD68; green, c-Maf), high-power field (×40)

Immunohistochemistry combining CD68 with antibodies specific for phospho-STAT1 or c-Maf revealed double-positive cells in all cases. The distinction between double-positive cells was obvious, with little or no background staining (Fig. 1). Heterogeneous expression in double-positive cells was observed in whole sections. Overall, we observed significantly higher numbers of CD68/c-Maf-positive cells (median 19, 1–156) than CD68/phospho-STAT1-positive cells (median 6, 1–111, *p* < 0.001). In addition, the expression level of PD-L1 in tumor cells ranged from 37 to 170, whereas that of tumor-infiltrating immune cells ranged from 34 to 173. Detailed results of the markers we examined are presented in Supplementary Table 2.

### Sensitivity and Specificity Based on Overall and Disease-Free Survival in Cohorts 1 and 2

We determined the cut-off values of positive cells to assess correlations with survival using receiver operating characteristic (ROC) curve analysis on the basis of the sensitivity and specificity of the relevant outcome for each positive cell count (Fig. S1, cohort 1). If a curve was generated from pairs of weighted mean sensitivities and specificities, discrimination of the program for survival was expressed by the area under the curve (Fig. S1). The cut-off values for CD68/phospho-STAT1- and CD68/c-Maf-positive cells were less than 5 or greater than 11, respectively, for determining overall survival (Fig. S1a and b). A cut-off value of 0.19 or less for the M1/M2 ratio was also determined for overall survival (Fig. S1c). Detailed data are presented in Supplementary Table 3; these cut-off values were generally appropriate.

Kaplan–Meier analyses were performed to determine the associations of disease-free and overall survival with each macrophage phenotype (cohort 2). Patients with low CD68/phospho-STAT1 expression had poorer disease-free and overall survival than patients with high CD68/phospho-STAT1 expression [*p* < 0.0001 (Fig. S2a, b)]. In addition, disease-free and overall survival were worse in patients with high CD68/c-Maf expression [DFS 0.0056*,* OS 0.0054 (Fig. S2c, d)] compared with low CD68/c-Maf expression. Patients with low M1/M2 ratios showed poor overall and disease-free survival (Fig. S2e, f).

### Associations of Clinicopathological Findings and Markers (M1 and M2) with Patient Survival in Cohort 2

For disease-free survival, eight factors (sex, smoking history, pathological stage, lymphatic invasion, venous invasion, STAS, M1-positive cells, M2-positive cells) were observed, and five (pathological stage, lymphatic invasion, STAS, M1-positive cells, M2-positive cells) were retained (Table 2). For overall survival, nine factors (sex, smoking history, age, pathological stage, lymphatic invasion, venous invasion, IASLC/ATS/ERS, M1-positive cells, M2-positive cells) were identified in univariate analysis. However, only pathological stage, M1-positive cells, and M2-positive cells were retained in multivariate analysis (Table 3).

**Table 2:** Association of clinicopathological variables, PD-L1, M1, and M2 with disease-free survival on univariate and multivariate analyses

Factor		Univariate analysis			Multivariate analysis		
		HR	(95% CI)	*p* Value	HR	(95% CI)	*p* Value
Sex	Male versus female	1.7	(1.17–2.48)	0.0054	1.23	(0.77–1.97)	0.3923
Smoke	Smoker versus never	1.76	(1.21–2.56)	0.0034	1.18	(0.72–1.94)	0.5033
Age (years)	≥ 70 versus < 70	1.06	(0.73–1.54)	0.7575			
pStage	II and III versus I	4.12	(2.80–6.06)	< 0.0001	2.66	(1.68–4.23)	< 0.0001
Lymphatic invasion	Positive versus negative	4.05	(2.65–6.17)	< 0.0001	1.98	(1.20–3.25)	0.0074
Venous invasion	Positive versus negative	2.59	(1.71–3.93)	< 0.0001	1.28	(0.80–2.04)	0.3026
STAS	Positive versus negative	2.29	(1.57–3.34)	< 0.0001	1.65	(1.10–2.48)	0.0153
IASLC/ATS/ERS classification subtypes	High group versus low and intermediate group	1.54	(0.99–2.4)	0.0582			
PD-L1 (TC)	Positive versus negative	1.07	(0.65–1.76)	0.7848			
PD-L1 (IC)	Positive versus negative	1.25	(0.76–2.05)	0.3755			
CD68^+^/p-STAT1^+^ (M1)	Low versus high	4.41	(2.89–6.73)	< 0.0001	4.68	(3.00–7.31)	< 0.0001
CD68^+^/c-Maf^+^ (M2)	High versus low	1.78	(1.17–2.71)	0.0069	2.38	(1.54–3.70)	0.0001

*PD-L1* programmed cell death 1-ligand 1, *M1* M1 macrophage, *M2* M2 macrophage, *HR* hazard ratio, *95% CI* 95% confidence interval, *pStage* pathological stage, *STAS* spread through air spaces, *IASLC* International Association for the Study of Lung Cancer, *ATS* American Thoracic Society, *ERS* European Respiratory Society, *TC* tumor cells, *IC* tumor-infiltrating immune cells, *p-STAT1* phospho-STAT1

**Table 3:** Association of clinicopathological variables, PD-L1, M1, and M2 with overall survival on univariate and multivariate analyses

Factor		Univariate analysis			Multivariate analysis		
		HR	(95% CI)	*p* Value	HR	(95% CI)	*p* Value
Sex	Male versus female	2.08	(1.31–3.30)	0.0019	1.48	(0.81–2.69)	0.2021
Smoke	Smoker versus never	2.02	(1.27–3.21)	0.003	1.29	(0.69–2.44)	0.4234
Age (years)	≥ 70 versus < 70	1.67	(1.05–2.65)	0.0303	1.39	(0.85–2.27)	0.1893
pStage	II and III versus I	3.66	(2.30–5.83)	< 0.0001	1.95	(1.12–3.39)	0.0177
Lymphatic invasion	Positive versus negative	3.7	(2.28–6.00)	< 0.0001	1.32	(0.68–2.55)	0.405
Venous invasion	Positive versus negative	2.01	(1.21–3.33)	0.007	1.06	(0.59–1.90)	0.8541
STAS	Positive versus negative	1.44	(0.90–2.28)	0.1246			
IASLC/ATS/ERS classification subtypes	High group versus low and intermediate group	2.49	(1.54–4.02)	0.0002	1.66	(0.93–2.96)	0.0846
PD-L1 (TC)	Positive versus negative	1.19	(0.67–2.12)	0.5654			
PD-L1 (IC)	Positive versus negative	1.23	(0.69–2.20)	0.4811			
CD68^+^/p-STAT1^+^ (M1)	Low versus high	11.67	(5.60–24.31)	< 0.0001	10.81	(5.07–23.03)	< 0.0001
CD68^+^/c-Maf^+^ (M2)	High versus low	2.07	(1.22–3.51)	0.0072	2.46	(1.42–4.26)	0.0013

*PD-L1* programmed cell death 1-ligand 1, *M1* M1 macrophage, *M2* M2 macrophage, *HR* hazard ratio, *95% CI* 95% confidence interval *pStage* pathological stage, *STAS* spread through air spaces, *IASLC* International Association for the Study of Lung Cancer, *ATS* American Thoracic Society, *ERS* European Respiratory Society, *TC* tumor cells, *IC* tumor-infiltrating immune cells, *p-STAT1* phospho-STAT1

### Association of Clinicopathological Findings and Markers (M1/M2) with Patient Survival in Cohort 2

Next, we examined the associations of clinicopathological findings with disease-free survival. Univariate analysis identified seven factors [sex, smoking history, pathological stage, lymphatic invasion, venous invasion, STAS, M1/M2 ratio (Table 4)]. Four (pathological stage, lymphatic invasion, STAS, M1/M2 ratio) were retained in multivariate analysis (Table 4). Similarly, eight factors (sex, smoking history, age, pathological stage, lymphatic invasion, venous invasion, IASLC/ATS/ERS, M1/M2 ratio) were identified, and four (pathological stage, lymphatic invasion, STAS, M1/M2 ratio) were associated with overall survival in univariate analysis (Table 5). Three factors (pathological stage, lymphatic invasion, M1/M2 ratio) were also retained in multivariate analysis (Table 5).

**Table 4:** Association of clinicopathological variables, PD-L1, and M1/M2 ratio with disease-free survival on univariate and multivariate analyses

Factor		Univariate analysis			Multivariate analysis		
		HR	(95% CI)	*p* Value	HR	(95% CI)	*p* Value
Sex	Male versus female	1.7	(1.17–2.48)	0.0054	1.21	(0.73–2.00)	0.4701
Smoke	Smoker versus never	1.76	(1.21–2.56)	0.0034	1.2	(0.71–2.02)	0.4902
Age (years)	≥ 70 versus < 70	1.06	(0.73–1.54)	0.7575			
pStage	II and III versus I	4.12	(2.80–6.06)	< 0.0001	2.77	(1.75–4.38)	< 0.0001
Lymphatic invasion	Positive versus negative	4.05	(2.65–6.17)	< 0.0001	2.48	(1.51–4.07)	0.0003
Venous invasion	Positive versus negative	2.59	(1.71–3.93)	< 0.0001	1.15	(0.73–1.81)	0.5402
STAS	Positive versus negative	2.29	(1.57–3.34)	< 0.0001	1.56	(1.04–2.36)	0.0326
IASLC/ATS/ERS classification subtypes	High group versus low and intermediate group	1.54	(0.99–2.4)	0.0582			
PD-L1 (TC)	Positive versus negative	1.07	(0.65–1.76)	0.7848			
PD-L1 (IC)	Positive versus negative	1.25	(0.76–2.05)	0.3755			
M1/M2 ratio	Low versus high	2.74	(1.88–3.99)	< 0.0001	2.78	(1.87–4.11)	< 0.0001

*PD-L1* programmed cell death 1-ligand 1, *M1* M1 macrophage, *M2* M2 macrophage, *HR* hazard ratio, *95% CI* 95% confidence interval, *pStage* pathological stage, *STAS* spread through air spaces, *IASLC* International Association for the Study of Lung Cancer, *ATS* American Thoracic Society, *ERS* European Respiratory Society, *TC* tumor cells, *IC* tumor-infiltrating immune cells

**Table 5:** Association of clinicopathological variables, PD-L1, and M1/M2 ratio with overall survival on univariate and multivariate analyses

Factor		Univariate analysis			Multivariate analysis		
		HR	(95% CI)	*p* Value	HR	(95% CI)	*p* Value
Sex	Male versus female	2.08	(1.31–3.30)	0.0019	1.62	(0.85–3.09)	0.1418
Smoke	Smoker versus never	2.02	(1.27–3.21)	0.003	1.27	(0.65–2.50)	0.4855
Age (years)	≥ 70 versus < 70 years	1.67	(1.05–2.65)	0.0303	1.51	(0.94–2.44)	0.091
pStage	II and III versus I	3.66	(2.30–5.83)	< 0.0001	2.53	(1.46–4.40)	0.001
Lymphatic invasion	Positive versus negative	3.7	(2.28–6.00)	< 0.0001	2.34	(1.26–4.33)	0.007
Venous invasion	Positive versus negative	2.01	(1.21–3.33)	0.007	0.98	(0.57–1.69)	0.9468
STAS	Positive versus negative	1.44	(0.90–2.28)	0.1246			
IASLC/ATS/ERS classification subtypes	High group versus low and intermediate group	2.49	(1.54–4.02)	0.0002	1.06	(0.60–1.85)	0.8462
PD-L1 (TC)	Positive versus negative	1.19	(0.67–2.12)	0.5654			
PD-L1 (IC)	Positive versus negative	1.23	(0.69–2.20)	0.4811			
M1/M2 ratio	Low versus high	3.95	(2.48–6.28)	< 0.0001	3.86	(2.37–6.29)	< 0.0001

*PD-L1* programmed cell death 1-ligand 1, *M1* M1 macrophage, *M2* M2 macrophage, *HR* hazard ratio, *95% CI* 95% confidence interval, *pStage* pathological stage, *STAS* spread through air spaces, *IASLC* International Association for the Study of Lung Cancer, *ATS* American Thoracic Society, *ERS* European Respiratory Society, *TC* tumor cells, *IC* tumor-infiltrating immune cells

### Association of PD-L1 Expression with Patient Outcome

We assessed the expression of PD-L1 in both tumor and tumor-infiltrating immune cells in LADs. PD-L1 stained well and consequently was clearly identified. However, no correlation of expression with outcome was found in the validation cohort (cohort 2). Expression values are presented in Supplementary Table 2.

### Association of 5-Year Survival with Low and High Expression Levels of M1 and M2 and the M1/M2 Ratio

We examined the association of the 5-year survival rate with low and high expression levels of M1 and M2 and the M1/M2 ratio. Detailed data are summarized in Supplementary Table 4.

Finally, we examined each factor that was retained in the univariate analysis of DFS, stratified by the presence and absence of venous invasion in the present study. As a result, lymphatic invasion was found to be a confounding factor that affects venous invasion (Fig. S3).

## Discussion

Macrophages are activated into M1 (classically activated) or M2 (alternatively activated) phenotypes.^8,12^ Immunostaining is often used to examine the roles of these phenotypes. Nitric oxide synthase 2, Toll-like receptor (TLR) 2, TLR4, CD80, and CD86 are used as M1 markers, whereas CD115, CD206, arginase 1, CD163, and CD301 are recognized as M2 markers.^17,24^ However, it remains difficult to distinguish M1 from M2 macrophages definitively,^24^ because they can also be expressed in other cells, including lymphocytes and leukocytes.^14,15,23^ Moreover, macrophages can express markers simultaneously owing to the continuum of phenotypes between M1 and M2.^14,15,24^ To overcome this, double immunostaining for specific and pan macrophage markers is necessary.^12^ Identification of M1 and M2 macrophages relies on the accurate selection of markers for differentiation.^12^ In this study, we used CD68/phospho-STAT1 for M1 macrophages and CD68/c-Maf for M2 macrophages.

In response to interferon, STAT1 is upregulated, and its phosphorylated form binds to the promoter region of interferon-stimulated genes.^25^ A predominance of STAT1 activation promotes M1 polarization of TAMs by increasing pro-inflammatory cytokines,^7,24^ suggesting that STAT1 may be an M1 marker. TAMs isolated from STAT1-knockout mice fail to induce T-cell responses.^7,24,25^ They lack arginase I activity, which reduces nitric oxide production by inducible nitric oxide synthase (iNOS).^25,26^ Taken together, these data indicate that STAT1 activation in TAMs may upregulate iNOS and arginase I activity, resulting in T-cell activation.^25^ In this study, low expression of the CD68/phospho-STAT1-positive TAM phenotype was an independent factor predicting poor prognosis. In a previous study, STAT1 expression in macrophages identified patients with improved survival and an intact tumor immune system, who may benefit from immunotherapy.^25^ Thus, our findings contribute to elucidation of the role of TAM1 (M1) infiltration in the invasive tumor area.c-Maf is essential for macrophage self-renewal but is also expressed in T cells, including Th2 and Th17 cells.^27–29^ Liu et al. identified c-Maf as an essential regulator of immunosuppressive macrophage polarization^27^ and showed that c-Maf is predominantly expressed in M2-like macrophages in both mice and humans.^27^ Furthermore, inhibition of c-Maf in macrophages results in an M1-like phenotype with diminished immunosuppressive function, and promotes antitumor T-cell immunity, leading to significantly reduced tumor progression.^27^ Thus, c-Maf is a core molecule in immunosuppressive macrophage polarization. In this study, we showed for the first time that a high number of CD68/c-Maf -positive macrophages was correlated with prognosis in patients with LAD, similar to the findings of a previous report in cervical cancer.^28^

In this study, PD-L1 expression in cancer cells and tumor-infiltrating immune cells was not correlated with survival, suggesting that PD-L1 expression cannot predict outcomes in patients with LAD. Although cancer immunotherapy with anti-PD-L1 immune checkpoint inhibitors is widely used in patients with non-small cell lung cancer, many are resistant.^29–33^ Inhibition of c-Maf may contribute to overcoming resistance.^27^ Indeed, anti-PD-1 therapy combined with c-Maf inhibition significantly reduced tumor progression.^27^ Immunomodulators that specifically target c-Maf in macrophages may be promising, because c-Maf is a critical transcription factor for many immune cell subsets.^27^ Targeting patients with high numbers of c-Maf-positive macrophages may offer a novel strategy to reinforce current cancer immunotherapies.

Quantifying the M1/M2 ratio in TAMs could be used to evaluate macrophage polarization for clinical applications.^34^ The M1/M2 ratio is a more biologically relevant indicator of cancer prognosis than M1 or M2 density alone,^34,35^ and could represent either a positive or negative impact on tumor growth. A lower M1/M2 ratio often indicates a poor prognosis, whereas a better prognosis is associated with a higher M1/M2 ratio.^34–36^ In this study, we found an M1/M2 ratio of 0.19, suggesting that at diagnosis, the tumor microenvironment is polarized toward an M2 phenotype.^34^ These data are consistent with previous studies suggesting that M2 macrophages are involved in cancer development in several human malignancies, including cervical cancer.^28^ Further studies are needed to evaluate the mechanisms related to this macrophage phenotype ratio.

Vascular invasion is a well-known prognostic factor in LADs. A previous study showed that vascular invasion was an important factor that increased the likelihood of recurrence. However, no correlation of patient outcome with vascular invasion in LADs was found in the present study, likely because vascular invasion was found to be a confounding factor of lymphatic invasion. On the other hand, standardization of vascular invasion is important for assessing vascular invasion accurately. As HE staining alone is insufficient for evaluating vascular invasion, specific staining is necessary. A further prospective study with a larger cohort may be needed to clarify whether vascular invasion is a prognostic factor in LADs.

This study had some limitations. First, histological heterogeneity of M1 and M2 phenotypes may make it difficult to evaluate the expression patterns of macrophage-specific proteins. The specialization of macrophages in microenvironments explains their heterogeneity. Moreover, the heterogeneous functional properties of macrophages could result from their locations in tumor tissues.^37,38^ Although it is difficult to determine the tumor center and invasive front in the majority of LADs, which often exhibit a lepidic pattern at the periphery, the following explanation may apply. There are three typical locations: the tumor center, invasive front, and tumor stroma in gastric cancer.^37^ According to the relative macrophage densities in the tumor nest or stroma, gastric cancer cases could be divided into nest-dominant and stroma-dominant patterns.^37^ Patients with a stroma-dominant pattern tend to have poorer survival and higher malignancy, and macrophages accumulating in the tumor stroma may participate more actively in stroma activation.^37^ Second, we did not validate the findings in a second cohort, because the first cohort was large. However, we believe that the current cohort was an adequate size for evaluating outcomes in patients with LAD. Finally, triple-stained CD68/phospho-STAT1/c-Maf cells should have been examined in each case, as results may have enabled evaluation of the role of infiltrating M1 and M2 within the tumor. However, it may be difficult to find appropriate specimens for triple immunostaining among old specimens (> 5 years after surgery). In addition, overlaying different colors in the same site (cytoplasm/cytoplasm or nucleus/nucleus) complicates immunohistochemical assessment. Although triple immunostaining was not examined in this study, we believe that double immunostaining was sufficient to evaluate infiltrating M1 and M2.

In conclusion, our data suggest that high CD68/c-Maf expression and low CD68/phospho-STAT1 expression are predictors of worse prognosis in patients with LAD. A low M1/M2 ratio may contribute to prediction of outcomes, suggesting that a shift from the M1 to the M2 phenotype in TAMs may be related to worse survival. Double immunostaining with these markers may be suitable for identifying M1 and M2 phenotypes in histological samples.

### Supplementary Information

Below is the link to the electronic supplementary material.

Supplementary file1 (TIF 199 kb)

Supplementary file2 (TIF 263 kb)

Supplementary file3 (TIF 4438 kb)

Supplementary file4 (DOCX 20 kb)

## Data Availability

The datasets used and/or analyzed during the current study are available from the corresponding author on reasonable request.
