# Supplementary material for: Prognostic Impact of Tumor-Associated Macrophage-Related Markers in Patients with Adenocarcinoma of the Lung
Source: Ann Surg Oncol. 2023 Jun 6;30(12):7527–37. doi: 10.1245/s10434-023-13384-9 (PMC10562267; doi:10.1245/s10434-023-13384-9)
Supplement: Supplementary file 4 — Supplementary file4 (DOCX 20 kb) [file 10434_2023_13384_MOESM4_ESM.docx]

Supplementary Table 1. Antibodies used in this study.

| Antibody target | Clone | Source | Dilution | Treatment |
| --- | --- | --- | --- | --- |
| CD68 | PG-M1 | Dako | Ready to use | None |
| Phospho-STAT1 | 58D6 | CST | 1:50 | Heat retrieval (pH 9.0) |
| c-Maf | EPR16484 | Abcam | 1:100 | Heat retrieval (pH 9.0) |
| PD-L1 | 22C3 | Dako | Ready to use | Heat retrieval (pH 6.0) |

Phospho-STAT1, phosphorylated signal transducer and activator of transcription 1; CST, Cell Signaling Technology; PD-L1, programmed cell death 1-ligand 1.

Supplementary Table 2. Expression of PD-L1 and M1, M2, and the M1/M2 ratio in cohort 2.

| Factor | Cohort 2 | |
| --- | --- | --- |
| Total | 207 |  |
| PD-L1 (TC) |  |  |
| Positive (%) | 37 | (17.9) |
| Negative (%) | 170 | (82.1) |
| PD-L1 (IC) |  |  |
| Positive (%) | 34 | (16.4) |
| Negative (%) | 173 | (83.6) |
| CD68+/p-STAT1+ (M1) |  |  |
| High (%) | 104 | (50.2) |
| Low (%) | 103 | (49.8) |
| CD68+/c-Maf+ (M2) |  |  |
| High (%) | 135 | (65.2) |
| Low (%) | 72 | (34.8) |
| M1/M2 ratio |  |  |
| High (%) | 135 | (65.2) |
| Low (%) | 72 | (34.8) |

PD-L1, programmed cell death 1-ligand 1; TC, tumor cells; IC, tumor-infiltrating immune cells; M1, M1 macrophage; M2, M2 macrophage; p-STAT1, phospho-STAT1.

Supplementary Table 3. Sensitivity, specificity, positive predictive value, negative predictive value, positive likelihood ratio, and negative likelihood ratio of CD68+/p-STAT1+ (M1) and CD68+/c-Maf+ (M2), CD68+/p-STAT1+ (M1) /CD68+/c-Maf+ (M2) ratio for overall survival.

|  | Sensitivity | Specificity | Positive predictive value | Negative predictive value | Positive likelihood ratio | Negative likelihood ratio |
| --- | --- | --- | --- | --- | --- | --- |
| CD68+/p-STAT1+ (M1) | 0.82 | 0.67 | 0.56 | 0.88 | 2.47 | 0.26 |
| CD68+/c-Maf+ (M2) | 0.91 | 0.42 | 0.45 | 0.9 | 1.58 | 0.21 |
| CD68+/p-STAT1+ (M1)/ CD68+/c-Maf+ (M2) ratio | 0.82 | 0.73 | 0.61 | 0.89 | 3.02 | 0.24 |

p-STAT1, phosphorylated signal transducer and activator of transcription 1; M1, M1 macrophage; M2, M2 macrophage.

Supplementary Table 4. 5-year survival rates with low and high expression levels of M1, M2 and the M1/M2 ratio.

|  | 5 years survival rate (%) | |
| --- | --- | --- |
| Factor | DFS | OS |
| CD68+/p-STAT1+ (M1) |  |  |
| High | 71.2 | 92.3 |
| Low | 21.4 | 34 |
| CD68+/c-Maf+ (M2) |  |  |
| High | 40 | 57 |
| Low | 58.3 | 75 |
| M1/M2 ratio |  |  |
| High | 60 | 78.5 |
| Low | 20.8 | 34.7 |

M1, M1 macrophage; M2, M2 macrophage; DFS, disease-free survival; OS, overall survival; p-STAT1, phospho-STAT1.
